# Lipid metabolism characterization in gastric cancer identifies signatures to predict prognostic and therapeutic responses

**DOI:** 10.3389/fgene.2022.959170

**Published:** 2022-11-03

**Authors:** Jiawei Zeng, Honglin Tan, Bin Huang, Qian Zhou, Qi Ke, Yan Dai, Jie Tang, Bei Xu, Jiafu Feng, Lin Yu

**Affiliations:** ^1^ Department of Clinical Laboratory, Mianyang Central Hospital, School of Medicine, University of Electronic Science and Technology of China, Mianyang, China; ^2^ Development and Regeneration Key Lab of Sichuan Province, Department of Histology and Embryology, Chengdu Medical College, Chengdu, China; ^3^ Emergency Department, Mianyang Central Hospital, School of Medicine, University of Electronic Science and Technology of China, Mianyang, China; ^4^ Department of Pathology, Mianyang Central Hospital, School of Medicine, University of Electronic Science and Technology of China, Mianyang, China; ^5^ Department of Ophthalmology, Mianyang Central Hospital, School of Medicine, University of Electronic Science and Technology of China, Mianyang, China; ^6^ NHC Key Laboratory of Nuclear Technology Medical Transformation, (Mianyang Central Hospital), Mianyang, China

**Keywords:** gastric cancer, LMscore, therapeutic response, sphingolipid metabolism, lipidome pseudotargeted metabolomics

## Abstract

**Purpose:** Increasing evidence has elucidated the significance of lipid metabolism in predicting therapeutic efficacy. Obviously, a systematic analysis of lipid metabolism characterizations of gastric cancer (GC) needs to be reported.

**Experimental design:** Based on two proposed computational algorithms (TCGA-STAD and GSE84437), the lipid metabolism characterization of 367 GC patients and its systematic relationship with genomic characteristics, clinicopathologic features, and clinical outcomes of GC were analyzed in our study. Differentially expressed genes (DEGs) were identified based on the lipid metabolism cluster. At the same time, we applied single-factor Cox regression and random forest to screen signature genes to construct a prognostic model, namely, the lipid metabolism score (LMscore). Next, we deeply explored the predictive value of the LMscore for GC. To verify the specific changes in lipid metabolism, a total of 90 serum, 30 tumor, and non-tumor adjacent tissues from GC patients, were included for pseudotargeted metabolomics analysis *via* SCIEX triple quad 5500 LC-MS/MS system.

**Results:** Five lipid metabolism signature genes were identified from a total of 3,104 DEGs. The LMscore could be a prognosticator for survival in different clinicopathological GC cohorts. As well, the LMscore was identified as a predictive biomarker for responses to immunotherapy and chemotherapeutic drugs. Additionally, significant changes in sphingolipid metabolism and sphingolipid molecules were discovered in cancer tissue from GC patients by pseudotargeted metabolomics.

**Conclusion:** In conclusion, multivariate analysis revealed that the LMscore was an independent prognostic biomarker of patient survival and therapeutic responses in GC. Depicting a comprehensive landscape of the characteristics of lipid metabolism may help to provide insights into the pathogenesis of GC, interpret the responses of gastric tumors to therapies, and achieve a better outcome in the treatment of GC. In addition, significant alterations of sphingolipid metabolism and increased levels of sphingolipids, in particular, sphingosine (d16:1) and ceramide, were discovered in GC tissue by lipidome pseudotargeted metabolomics, and most of the sphingolipid molecules have the potential to be diagnostic biomarkers for GC.

## 1 Introduction

To the best of our knowledge, gastric cancer (GC) is still the second leading cause of death from the malignant disease worldwide and has remained relatively unchanged in the past few decades (Takahashi et al., 2013; Wang et al., 2019). Obviously, there is an urgent need to improve the survival rate of GC patients. Metabolic reprogramming, especially altered lipid metabolism, has been identified to support the requirements of many different cancer cells for exponential growth and proliferation (Park et al., 2018; Bian et al., 2020; Faubert et al., 2020). Due to its biological significance in the survival, signal transduction, and therapeutic response of cancer cells, lipid metabolism characterizations have important therapeutic implications (Martinez-Outschoorn et al., 2017; Tang et al., 2021). The efficacy of drugs targeting lipogenic enzymes, exogenous lipid uptake, inflammatory signaling pathways, etc, have been evaluated in preclinical and clinical studies (Fernandez et al., 2020).

In our previous study, we observed altered lipid metabolism based on the serum levels of patients with chronic gastritis and GC *via* untargeted metabolomics. Meanwhile, the results of fasting lipid profile analysis were significantly different in the control, chronic gastritis, and GC group (*p* < 0.05). Thus, these results indicated that the characteristics of lipid metabolism may influence the development of GC (Yu et al., 2021). In line with other studies, more lipid metabolism-related signaling pathways were validated *in vitro* and *in vivo* in GC. For instance, PHTF2 was significantly enriched in the fatty acid metabolism pathway and can regulate lipid metabolism to affect GC tumorigenesis (Chi et al., 2020). Lipid metabolism-related proteins, such as acyl-CoA thioesterase one and GLI family zinc finger 3, were highly expressed in GC (Wang et al., 2018). CD36 was validated as a mediator of GC metastasis by regulating lipid metabolism through the AKT/GSK-3β/β-catenin signaling pathway (Pan et al., 2019). However, the associations between lipid metabolism and the pathological development of GC still remain confusing.

To research the relationship between lipid metabolism and clinical prognosis in GC, we applied two proposed computational algorithms to estimate the lipid metabolism patterns of 1,536 tumors from GC patients (768 for immune infiltration, 431 for GEO, and 337 for TCGA) and systematically study the correlation among the lipid metabolism phenotypes with genomic characteristics, clinicopathologic features and clinical outcomes of GC. Finally, lipidome pseudotargeted metabolomics was applied to validate the specific change of lipid metabolism in GC patients.

## 2 Materials and methods

### 2.1 Materials and methods for two proposed computational algorithms

#### 2.1.1 Gastric cancer datasets and data processing

GC gene expression datasets were publicly available and reported with full clinical annotations. Patients with overall survival less than 30 days were removed from further evaluation, and cancer samples were available to construct the LMscore. The transcriptomic dataset (https://gdc.xenahubs.net/download/TCGA-STAD.htseq_fpkm.tsv.gz), the annotation data (https://gdc.xenahubs.net/download/gencode.v22.annotation.gene.probeMap), the phenotypic data (https://gdc.xenahubs.net/download/TCGA-STAD.GDC_phenotype.tsv.gz), the MSigdb lipid metabolism gene set (http://www.gsea-msigdb.org/gsea/msigdb/cards/REACTOME_METABOLISM_OF_LIPIDS), the mutation data (https://gdc.cancer.gov/about-data/publications/mc3-2017/mc3.v0.2.8.PUBLIC.maf.gz) and the CNV data of GC (https://gdc-hub.s3.us-east-1.amazonaws.com/download/TCGA-STAD.cnv.tsv.gz) were processed according to previous studies (Zeng et al., 2019; Chen et al., 2021). The microarray data of GSE84437 generated by Affymetrix were obtained from the Gene Expression Omnibus (GEO), included 768 GC specimens and 626 lipid metabolism genes for subsequent analysis. Information about the GC datasets were detailed in Supplementary Table S1.

#### 2.1.2 Differentially expressed genes associated with lipid metabolism

To identify genes associated with lipid metabolism, we grouped patients into lipid metabolism clusters and lipid metabolism gene clusters. Differentially expressed genes (DEGs) in different groups were determined by the limma package (Ritchie et al., 2015). Then, the ggpubr package was used to show the differentially expressed lipid metabolism genes in normal and cancer samples, and the RCircos package was used to display DEGs at chromosomal positions. Finally, we applied packages of R factoextra and FactoMineR in principal component analysis (PCA) for DEGs to characterize the classification of samples. Tumors with qualitatively different lipid metabolism cell infiltration patterns were grouped using hierarchical agglomerative clustering. Unsupervised clustering methods were used to identify lipid metabolism patterns and classify patients for further analysis. A consensus clustering algorithm was applied to determine the number of clusters in the GC cohort to assess the stability of the discovered clusters. This procedure was performed using the ConsensuClusterPlus package and was repeated 100 times to ensure the stability of the classification.

#### 2.1.3 Generation of lipid metabolism gene signatures

The construction of lipid metabolism metagenes was performed as previous study (Zeng et al., 2019; Jiang et al., 2021). Based on the differential genes in the lipid metabolism cluster, we firstly used single factor regression analysis to screen out genes with prognostic differences, with a threshold of *p* < 0.001, and then used random forest to extract characteristic genes with a Gini index >10. Then, principal component analysis (PCA) was performed, and principal components 1 and 2 were extracted to serve as the signature score. After obtaining the prognostic value of each gene signature score, we defined the LMscore of each patient as follows:

LMscore=∑(PC1i+PC2i)

where i is the expression level of gene i. The cutoff values of each dataset were evaluated based on the association between patient overall survival and the LMscore in each separate dataset using the survival and survminer packages, and patients were then divided into low and high LMscore groups.

#### 2.1.4 Predictive value of the lipid metabolism score to estimate therapeutic effect

The immune gene set comes from the ImmPort database and the InnateDB database (Breuer et al., 2013; Bhattacharya et al., 2018). Tumor immune dysfunction and exclusion (TIDE) was used to investigate the predictive value of the LM score for immunotherapy, and the TIDE score was shown in the high- and low-risk groups (Jiang et al., 2018). The pRRophetic R package was used to determine whether the LMscore could accurately predict clinical chemotherapeutic responses (Geeleher et al., 2014).

### 2.2 Materials and methods for lipidome pseudotargeted metabolomics

#### 2.2.1 Study participants for lipidome pseudotargeted metabolomics

A total of 90 serum (30 control individuals, 30 CG patients and 30 GC patients), as well as 30 tumor and 30 adjacent tissues from the GC patients, were gathered and weighed for pseudotargeted metabolomics analysis, which was approved by the Ethics Committee of Mianyang Central Hospital. All sample were collected after the routine inspection. Meanwhile, the inclusion criteria and exclusion criteria for the disease groups were similar to the reported literature (Yu et al., 2021). All serum samples were collected after fasting. The detailed clinicopathological characteristics are shown in Table 1.

**TABLE 1:** Clinical characteristics of the subjects.

Group	Control (*n* = 30)	CG (*n* = 30)	GC (*n* = 30)	*χ* ^*2*^ */F* value	*p*
Male/female (n)	15/15	15/15	23/7	5.875	0.053
Age (years ±standard deviation)	52 ± 9.03	55.14 ± 7.65	63.57 ± 9.17^*#^	13.436	0.000
History					
*H. pylori* infection	0	3	0	NA	NA
Gastritis (n)	0	15	NA	NA	NA
Chronic active gastritis (n)	0	12	NA	NA	NA
Intestinal metaplasia and/or atrophy (n)	0	0	NA	NA	NA
Endoscopic diagnosis					
Normal	NA	0	NA	NA	NA
Hiatal hernia	NA	0	NA	NA	NA
Esophagitis	NA	0	NA	NA	NA
Antroduodenitis	NA	0	NA	NA	NA
Duodenal ulcer	NA	0	NA	NA	NA
Gastric ulcer	NA	10	NA	NA	NA
Other	NA	20	NA	NA	NA
Tumor localization					
Antrum	NA	NA	9	NA	NA
Corpus	NA	NA	8	NA	NA
Cardias	NA	NA	3	NA	NA
Unknown	NA	NA	10	NA	NA
Histologic grade					
Grade 1	NA	NA	18	NA	NA
Grade 2	NA	NA	5	NA	NA
Grade 3	NA	NA	1	NA	NA
Grade 4	NA	NA	6	NA	NA
TNM stage					
I	NA	NA	6	NA	NA
II	NA	NA	12	NA	NA
III	NA	NA	5	NA	NA
IV	NA	NA	1	NA	NA
Unknown	NA	NA	6	NA	NA

Compared with the control group, *p < 0.05. Compared with the CG, group, ^#^ *p* < 0.05.

#### 2.2.2 Lipidome pseudotargeted metabolomics analysis

According to the criteria, serum sample and tissue sample were separately prepared for lipidome pseudotargeted metabolomics analysis. The strategy for lipidome pseudotargeted metabolomics analysis have been reported (Chen et al., 2013; Li et al., 2020). Firstly, the information of the characteristic ion pairs (the retention time, primary mass spectrum, and secondary mass spectrum data) were from serum untargeted metabolomics analysis. Next, the multiple reaction monitoring (MRM) mode was adopted to perform lipidome pseudotargeted metabolomics analysis for tissue sample. ONE-MAP was used for lipidome pseudotargeted metabolomics data analysis (Bro and Smilde, 2014).

### 2.3 Statistical analysis

For comparisons of two groups, statistical significance for normally distributed variables was estimated by unpaired Student’s *t*-tests. For comparisons of more than two groups, Kruskal-Wallis tests were used as nonparametric methods. Correlation coefficients were computed by Spearman and distance correlation analyses. Two-sided Fisher exact tests were used to analyze contingency tables. The cutoff values of each dataset were evaluated based on the association between patient overall survival and the LMscore in each separate dataset using the survminer package. The forest plot package was used to present the results of group analysis for the LMscore in GC datasets and TCGA datasets. The Kaplan-Meier method was used to generate survival curves for the groups in each dataset, and the log-rank test was used to determine the statistical significance of differences (Bland and Altman, 1998). The hazard ratios for univariate analysis was calculated by a univariate Cox proportional hazards regression model. A multivariate Cox regression model was used to determine independent prognostic factors using the survminer package. All statistical analyses were conducted using R or SPSS software, and the *p* values were two-sided. *p* < 0.05 was considered statistically significant.

## 3 Results

### 3.1 Characterization of lipid metabolism in gastric cancer and distinct patterns of lipid metabolism subtypes

**FIGURE 1:**
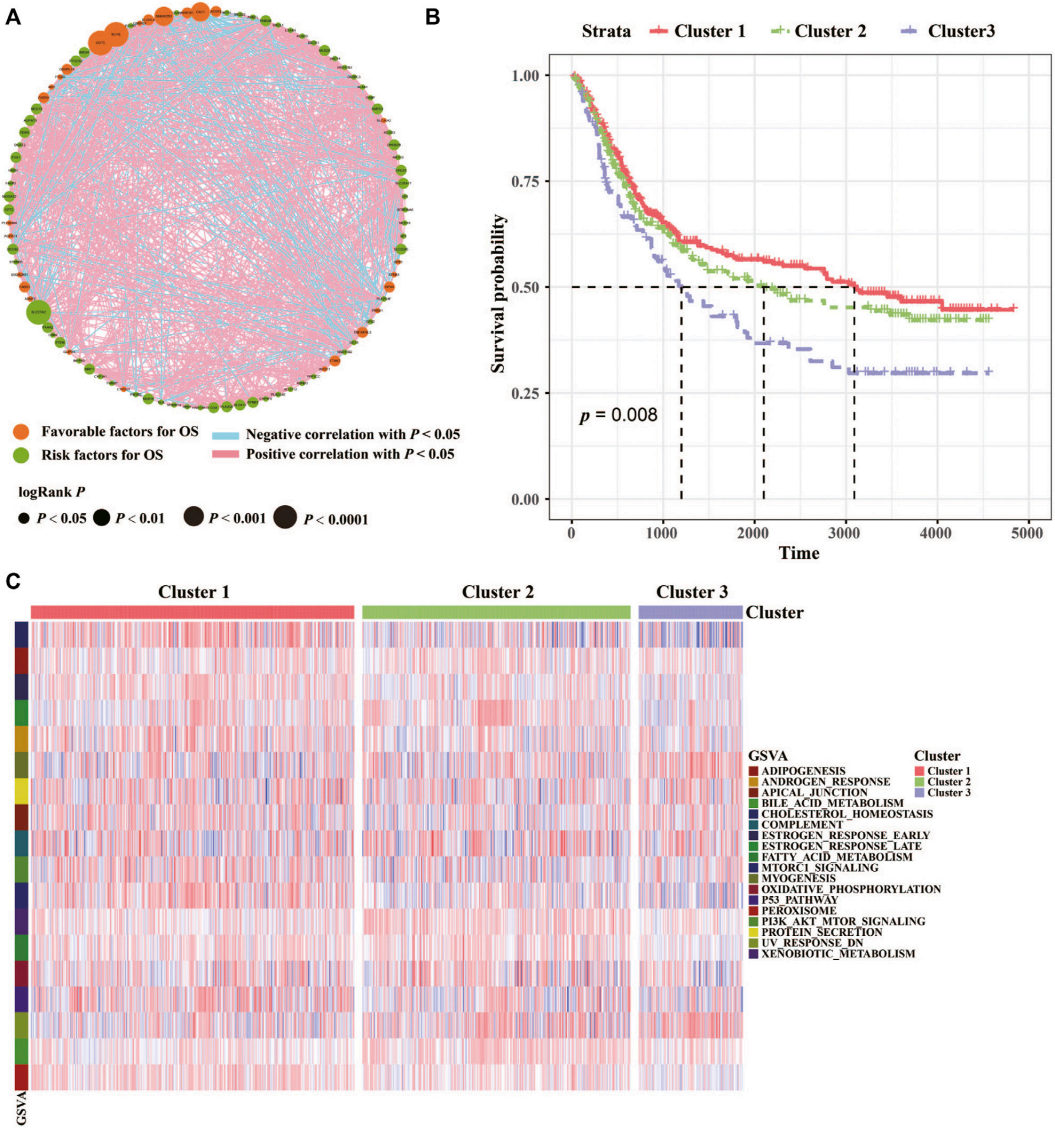
Characterizations of lipid metabolism in GC. **(A)** Cellular interactions of lipid metabolism in GC. The size of each cell represents the survival impact of each lipid metabolism gene. Favorable factors for OS, green circle; Risk factors for OS, orange circle. Positive correlation, red square; Negative correlation, blue square. **(B)** The association between lipid metabolism patterns and OS of 768 patients in the TCGA and GEO datasets. And the log-rank test shows an overall *p* < 0.05. **(C)** GSVA (gene set variation analysis) between the three clusters. Cluster 1, red; Cluster 2, green; Cluster 3, purple.

The clinicopathological characteristics of the GC cohort in the present study were shown in Supplementary Table S1. The TCGA dataset comprised 499 patients, and their transcriptomic data, were included in our initial analysis. Differentially expressed lipid metabolism-related genes were determined by significance criteria (adjusted *p* value <0.05 and |logFC| ≥ 1) as implemented in the limma package. Then, the differential expression of lipid metabolism genes in normal and cancer samples were showed. To identify the optimal cluster number, clustering stability was assessed according to the ConsensusClusterPlus package. The consensus matrix supported the existence of three robust clusters for GC (data not shown). We identified 94 prognosis-related lipid metabolism genes through univariate Cox regression analysis, among which 27 were protective factors and 67 were risk factors (Figure 1A). The hmisc package was used to calculate the correlation of lipid metabolism genes with prognostic efficacy, and 7 differentially expressed and prognosis-related lipid metabolism genes were clustered into three categories. In addition, the three clusters had significant prognostic differences (*p* = 0.008) (Figure 1B). Finally, a total of 18 pathways were enriched based on the lipid metabolism gene expression profile by GSVA (gene set variation analysis) (Figure 1C).

### 3.2 Construction and validation of the lipid metabolism score in different gastric cancer datasets

**FIGURE 2:**
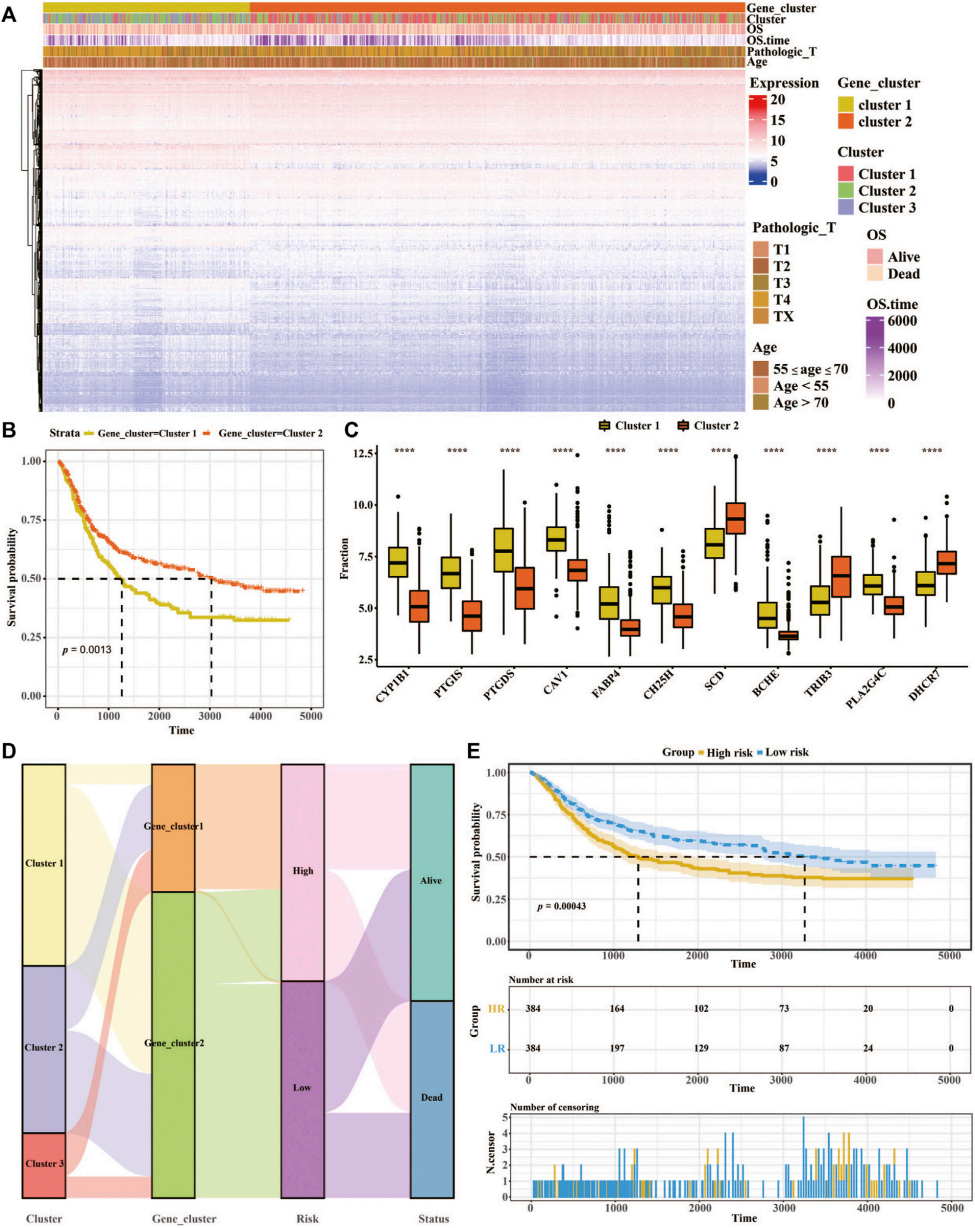
Constructing the prognostic model of the LMscore based on lipid metabolism signatures in GC. **(A)** Heatmap of unsupervised analysis and hierarchical clustering of the 3,104 DEGs and their associations with clinicopathological characteristics. **(B)** Kaplan-Meier plots for the association between lipid metabolism gene clusters and OS of 768 patients in the TCGA and GEO datasets. And the log-rank test shows an overall *p* = 0.0013. **(C)** The fraction of 11 differentially expressed genes in two lipid metabolism gene clusters. **(D)** Alluvial diagram showing the correlation of lipid metabolism subtypes with LMscore groups and clinical outcomes. **(E)** Kaplan-Meier curves of high- and low-LM score groups in the entire TCGA cohort. Log-rank test shows an overall *p* = 0.00043. *, *p* ≤ 0.05; **, *p* ≤ 0.01; ***, *p* ≤ 0.001; ****, *p* ≤ 0.0001, *p* ≤ 0.0001.

Analysis of K-means based onDEGs was then employed to classify the patients into two groups (Figure 2A). Survival analysis of patient and a total of 11 differentially expressed genes among the two lipid metabolism gene clusters were determined (Figures 2B,C). In addition, the relationships of lipid metabolism subtypes and LMscore groups as well as the outcomes of patients were analyzed. According to the median of the LMscore, and we found that overall survival was significantly longer for patients in the low LMscore group than for patients in the high LMscore group (Figure 2D). Moreover, the LMscore was established for each patient based on the prognostic value of each signature gene. As shown in Figure 2E, the LMscore could effectively distinguish significantly different OS in the entire cohort. Notably, patients with a low LMscore had significantly higher survival probability than those with a high LMscore (*n* = 384, *p* = 0.00043), which was validated in the GSE15459 dataset (Supplementary Figure S1).

We also studied the difference in the LMscore in the clinical groups using the GC cohort from TCGA and GEO databases. We found that the LMscore tended to increase as the tumor volume increased, which indicated that a high LMscore denotes a worse prognosis (Supplementary Figure S2A). There was a high incidence of GC with an average age of 33 years old. Interestingly, we also found that the LM score in the age <55 group was higher than that in the other two groups (55 ≤ age ≤70, and age >55) (Supplementary Figure S2B). When comparing the different grade, stage and lymph node groups, disease progression was associated with an increased LMscore. In addition, there were significant differences in the LMscore between the high and low lymph node number groups (Supplementary Figures S2C,D,E). Finally, the robustness of our model was proven, and the high LMscore group indeed had a worse prognosis (Supplementary Figure S3).

### 3.3 Predictive value of the lipid metabolism score as a biomarker for therapeutic effect in gastric cancer

**FIGURE 3:**
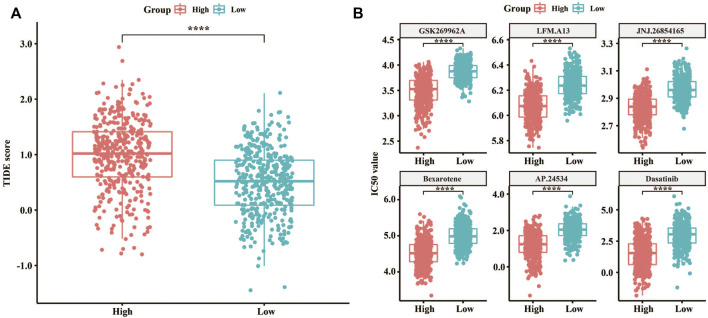
Predictive value of the LMscore as a biomarker for immunotherapy and chemotherapy for GC. **(A)** Box plots showing TIDE scores between the high and low LMscore groups. **(B)** Box plots showing the IC50 values between the high and low LMscore groups stratified by the drug, GSK269962A, and LFM. A13, JNJ.26854165, Bexarotene, AP.24534, and Dasatinib. ****, *p* < 2.22e-16.

TIDE was used to evaluate the LM score as a predictor of immunotherapy, and significant differences were observed in the TIDE score between the high and low LM groups (Figure 3A). Meanwhile, the TIDE prediction score was in the high LM group was significantly higher than that in the low LM group (*n* = 384, *p* < 2.22e-16), which indicated that the low LM score groups were more sensitive to immunotherapy (Jiang et al., 2018). Additionally, comparison of the IC50 of different drugs was applied in high and low LMscore groups, of which 67 kinds of drugs were significantly differential, and the high LMscore group had higher sensitivity. The first six drugs were displayed according to the *p* value (*p* < 2.22e-16) (Figure 3B). Totally, the difference of the predictive value of lipid metabolism between immunotherapy and chemotherapy, and the underlying molecular mechanism need to validate.3.4 Significant alterations of sphingolipid metabolism in GC discovered by lipidome pseudotargeted metabolomics.

**FIGURE 4:**
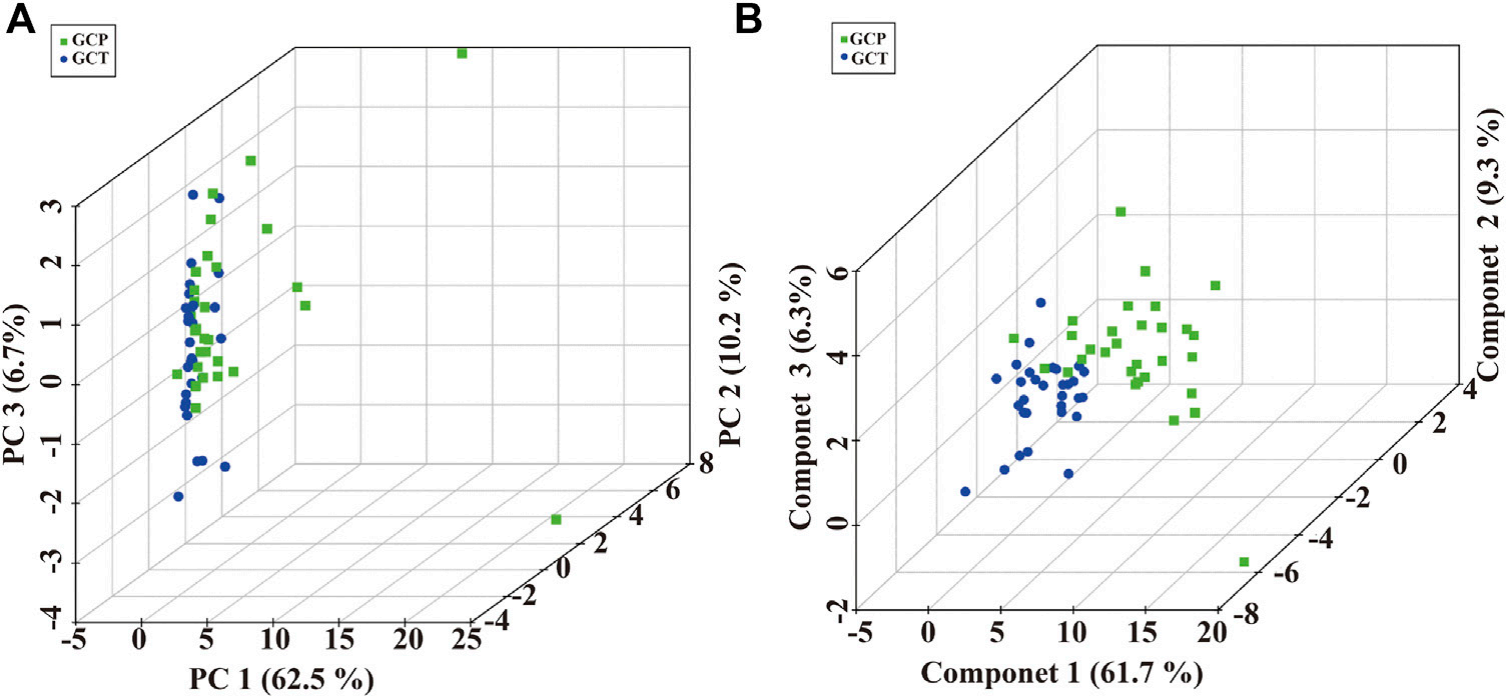
Multivariate analysis of pseudotargeted metabolomics data of tissue from the GC patients. **(A)** Three-dimensional score plot of the PCA models. **(B)** Score plots for the first three latent components of the PLS-DA model. Cancer tissue in GC patients, GCT; adjacent tissue in GC patients, GCP.

To validated our preliminary research, a total of 90 serum samples were firstly used for untargeted metabolomics analysis by SCIEX triple quad 5500 LC-MS/MS system. Representative total ion chromatograms (TIC) in positive and negative ion mode were selected and showed in Supplementary Tables S2, S3. Based on the result of untargeted metabolomics analysis, lipidome pseudotargeted metabolomics was determined to the metabolism characteristics of tissue from GC patients. Next, metabolomics data were analyzed by multivariate methods including principal component analysis (PCA) and partial least squares discriminant analysis (PLS-DA). Differentially accumulated metabolites were identified based on variable importance in the projection (VIP) > 1 in the loadings plot, FC > 1.5 or FC < 2/3, and *p* < 0.05. And, the AUC of the differential metabolites were calculated by the ROC analysis. PCA and PLS-DA were sufficient to characterize the results of tissue metabolite profiling (Figure 4). From the global overview of ion features, we interestingly found that significant alterations of sphingolipid metabolism were discovered by lipidome pseudotargeted metabolomics in cancer tissue from GC patients. In addition, sphingolipids molecules including sphingosine (d16:1) and the group of compounds known as ceramides, were validated in GC patients. According to the basic principle for ROC analysis (Hajian-Tilaki, 2013), differential metabolites were chosen as candidate markers when AUC >0.70 (Table 2).

**TABLE 2:** Differential metabolites in GC tumor tissue when compared to adjacent tissue.

Compounds	FC^a^	log2(FC)	VIP	AUC	CI	*p* (*U* test)	*p* (homogeneity variance test)
sphingosine (d16:1)	4.38	2.13	1.11	0.73	0.60–0.86	1.81E-03	2.07E-20
Cer (d18:1/20:0)	3.97	1.99	1.01	0.87	0.77–0.97	8.51E-07	2.86E-24
Cer (d18:0/18:0)	3.67	1.88	1.08	0.83	0.72–0.94	1.13E-05	6.14E-22
Cer (d18:2/21:0)	3.45	1.79	1.32	0.85	0.75–0.96	2.78E-06	3.62E-13
GlcCer(d18:1/12:0)	3.00	1.59	1.03	0.77	0.64–0.89	4.10E-04	4.37E-12
Cer (d18:2/20:0)	2.67	1.42	1.92	0.92	0.85–1.00	1.77E-08	6.32E-07
HexCer (d18:2/20:0)	2.64	1.40	1.36	0.86	0.77–0.95	1.58E-06	3.45E-03
Cer (d18:2/23:0)	2.13	1.09	1.12	0.74	0.61–0.87	1.48E-03	6.22E-06
SM 38:2	2.13	1.09	1.22	0.90	0.82–0.98	1.20E-07	1.24E-06
Cer (d18:2/18:0)	2.07	1.05	1.68	0.88	0.80–0.97	3.66E-07	5.31E-04
Cer (d18:2/22:0)	2.06	1.04	1.34	0.83	0.72–0.93	1.48E-05	2.61E-04
Cer (d18:0/24:1)	2.00	1.00	1.09	0.84	0.74–0.94	5.27E-06	9.58E-08
Cer (d16:1/22:0)	1.99	0.99	1.01	0.75	0.62–0.87	9.27E-04	2.81E-05
Cer (d18:1/24:0)	1.94	0.96	1.06	0.84	0.74–0.94	6.07E-06	1.33E-07
Cer (d18:1/23:1)	1.91	0.93	1.08	0.78	0.65–0.91	1.84E-04	3.66E-04
Cer (d18:1/24:1)	1.90	0.93	1.03	0.83	0.72–0.94	1.21E-05	2.48E-07
Cer (d18:0/16:0)	1.89	0.92	1.06	0.79	0.67–0.91	1.29E-04	3.40E-03
Cer (d18:2/24:1)	1.78	0.83	1.18	0.83	0.73–0.94	9.19E-06	7.03E-05

^a^ FC, FC > 1.5 indicates the upregulated level, FC < 2/3 indicates the downregulated level.

## 4 Discussion

Cancer cells possess altered cellular metabolism, which provides the biochemical basis to support tumorigenicity and malignancy, including in GC (Huang et al., 2020; Snaebjornsson et al., 2020). We previously found that altered lipid metabolism is the most prominent metabolic alteration in GC by untargeted metabolomics (Yu et al., 2021). The decrease in lipid profile in cancer patients may be owing to their increased utilization of lipids by neoplastic cells in membrane biogenesis. However, the changes in lipid metabolism and the correlation between lipid metabolism and clinical outcome in GC patients have been poorly studied.

The study was mainly focused on the prognostic of the low and high LMScore groups in different clinical models. As we known, the prognosis of GC is related to many factors, such as LAURENS typing, HER-2, PD-L1 expression, and so on. Based on LAURENS typing, GC is classified into intestinal type and diffuse type adenocarcinoma. Previous research has found that diverse metabolic pathways, including cholesterol homeostasis, glycolysis and fatty acid metabolism were dysregulated between the diffuse- and intestinal-subtypes. Recent genetic profiling research has led to the development of a new classification, namely the newly defined genomically stable subtype, which shares many cases with the histopathologically diffuse type (Ling et al., 2020; Suh et al., 2022). In addition, based on different cancer genomics research, different molecular functions and mutational signatures characteristic will be validated. Our study possibly provides an understanding of lipid metabolism in GC heterogeneity (Nam and Lee, 2022). Next, we performed an unsupervised clustering method to classify GC samples based on the proportions of 28 types of immune cells. The proportions of infiltrating immune cells among the three lipid metabolism subtypes were divided into high- and low-infiltration groups, and there was a significant difference in survival when the two groups had infiltrating activated CD4 T-cells and natural killer T-cells (Data not show). Lipid metabolites can indirectly regulate T-cell activation (Shyer et al., 2020). For instance, β-oxidation of *de novo* fatty acids is important for the differentiation of CD4^+^ regulatory T-cells (Lochner et al., 2015). Studies have proven that lipid metabolism can limit the cytotoxic machinery of NK cells *via* mTOR signaling (Michelet et al., 2018). As well, a recent study showed blockade of PD-L1 in GC influences lipid metabolism by increasing FAPB4/5 expression in CD8^+^ tissue-resident memory T-cells (Mabrouk et al., 2022). Thus, lipid metabolism has been an anticancer target.

Consensus clustering has been widely used in genomic studies. In this study, we used consensus clustering to estimate the optimal *K* value. We found that when *K* = 3, the consensus matrix was the crispest (Lancichinetti and Fortunato, 2012). Therefore, GC samples were classified into three clusters. Based on the DEGs among the three clusters, we used this criterion to determine the optimal *K* value and classify GC samples. Finally, two lipid metabolism subtypes, LMscore-high and LMscore-low, were identified based on this GC cohort, and the top five genes were *CAV1*, *PALM*, *PCDH7*, *C14orf132,* and *CEP55*. These DEGs have been validated to regulate lipid metabolism in cell renal cell carcinoma1 (Zhang et al., 2021), prostate cancer (Vykoukal et al., 2020) and colon cancer (Jeffery et al., 2016), which indicated that these DEGs may be potential prognostic biomarkers and therapeutic targets for GC.

Integrated analysis revealed that the LMscore may be a prognostic biomarker for GC, and that the LMscore showed a positive correlation with the progression of GC. Our data also revealed that patients with higher histologic grade, higher clinical stage and more lymph nodes exhibited higher LMscore (Supplementary Figures S2,S3). Increasing evidence has indicated that enhanced synthesis or uptake of lipids contributes to rapid cancer cell growth and drug resistance. Besides, the IC50 values for the high LMscore group were lower than those of the low LMscore groups, which also demonstrated the different underlying molecular mechanism.

Therefore, the specific changes in lipid metabolism should be further verified. Here, we performed lipidome pseudotargeted metabolomics to investigate the metabolic features for GC. The detailed clinicopathological characteristics, such as age, gender, endoscopic diagnosis, esophagitis, tumor localization, histologic grade, and TNM stage were shown in Table 1. From the lipidome pseudotargeted metabolomics analysis, sphingolipid metabolism was significantly altered in the cancer tissue compared with adjacent tissue in GC patients. In addition, a group of sphingolipids molecules were validated in GC patients may be used as candidate biomarkers for GC diagnosis. Sphingosine (d16:1), SM 38:2, and series of ceramides including Cer (d18:0/18:0), Cer (d18:2/21:0), GlcCer (d18:1/12:0), and so on, were significantly upregulated (FC > 1.5) in cancer tissue from GC patients when compared with those in healthy subjects (Table 2). Sphingolipids have been linked to cancer drug resistance (Giussani et al., 2014; Ogretmen, 2018; Companioni et al., 2021). Besides, sphingolipid metabolism has been validated to regulate the susceptibility to host immune cells, and a comprehensive landscape of tumor microenvironment characteristics helps interpret the responses of GC to immunotherapies (Church and Galon, 2015; Popel, 2020). Therefore, the comprehensive evaluation of lipid metabolism characterization is complementary to better maximize the antitumor efficacy both immunotherapy and chemotherapy (Binnewies et al., 2018). Meanwhile, this study provided clues to explore the detailed molecular mechanism of sphingolipid metabolism in GC.

## 5 Conclusion

In conclusion, our present study depicts a comprehensive landscape of lipid metabolism signatures in GC. Meanwhile, the LMscore was proven to be a promising predictor of patient survival and therapeutic responses in GC, which might be helpful to improve therapy. Notably, our study is also the first to demonstrate the levels of sphingolipid metabolismare significantly different in GC tumor tissue when compared to adjacent tissue *via* lipidome pseudotargeted metabolomics. Additionally, differential sphingolipid molecules validated in this study may be as diagnostic biomarkers for the diagnosis and classification of GC.

## Data Availability

The bioinformatics analysis data are available from the corresponding author upon reasonable request. At the same time, the pseudotargeted metabolomics data presented in the study are deposited in the iProX partner repository, accession number PXD034233, which can be found below: http://proteomecentral.proteomexchange.org/cgi/GetDataset?ID=PXD034233.
