# Supplementary figures and images for: Lipid metabolism characterization in gastric cancer identifies signatures to predict prognostic and therapeutic responses
Source: Front Genet. 2022 Nov 3;13:959170. doi: 10.3389/fgene.2022.959170 (PMC9669965; doi:10.3389/fgene.2022.959170)

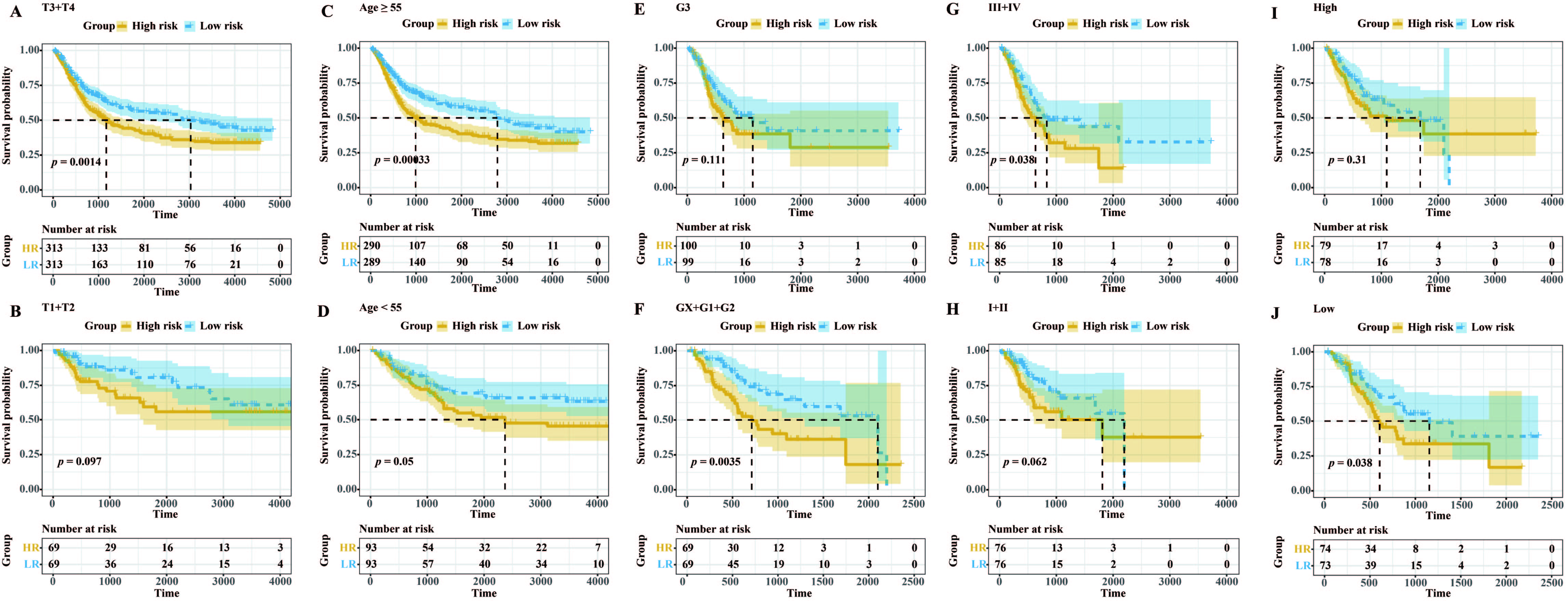

Supplement: Supplementary file 1 [file Image3.JPEG]

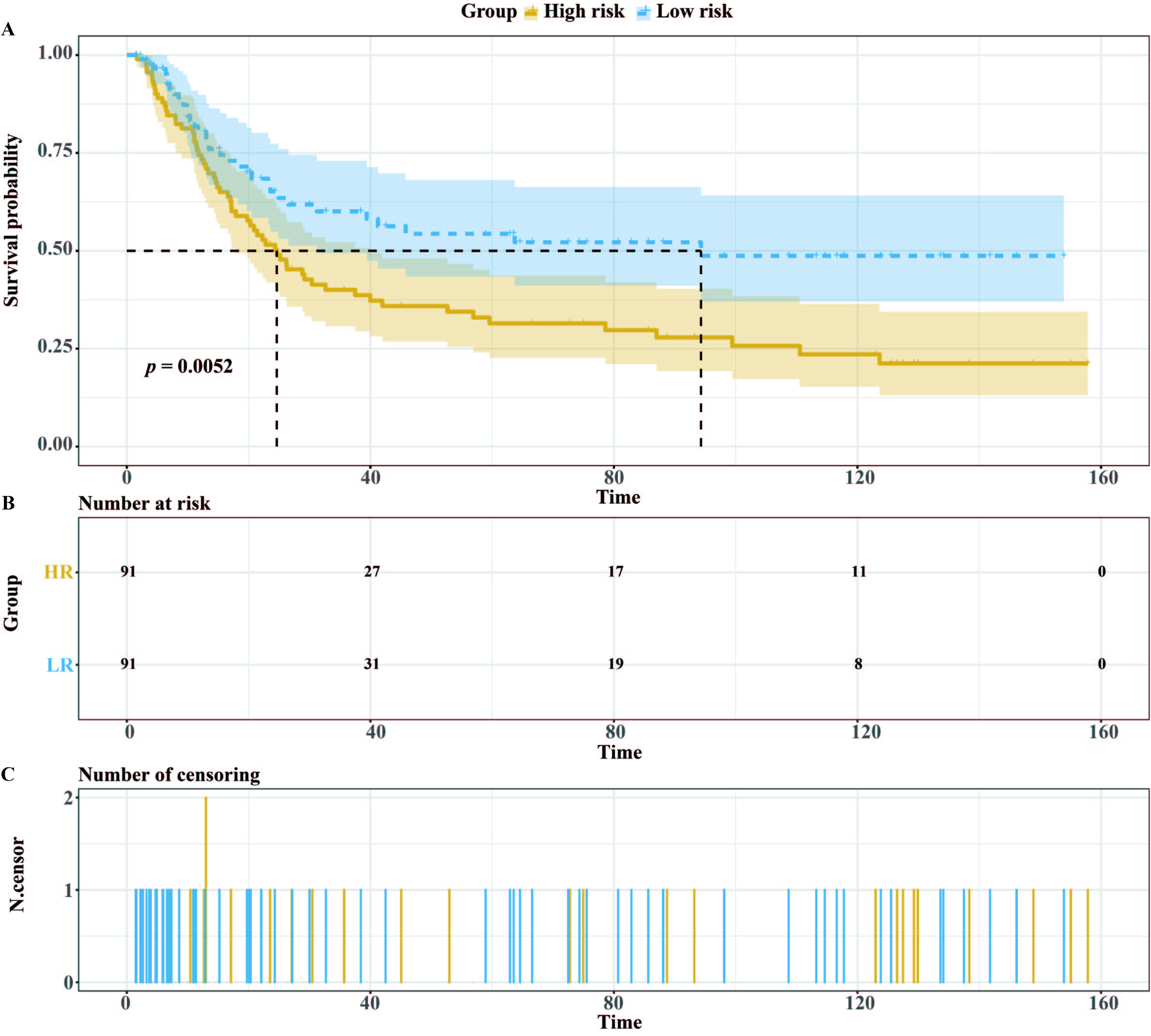

Supplement: Supplementary file 2 [file Image1.JPEG]

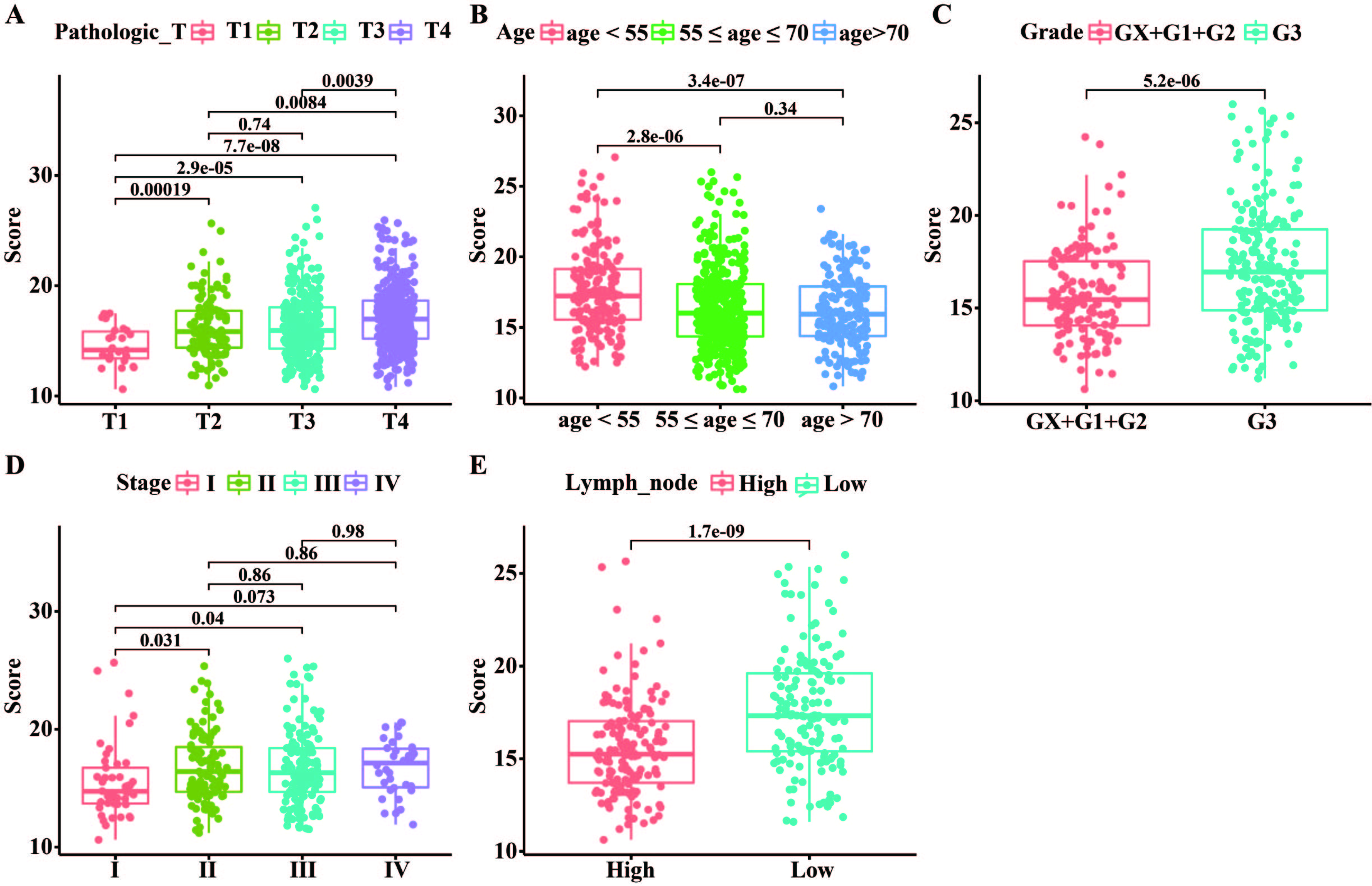

Supplement: Supplementary file 3 [file Image2.JPEG]
