# Supplementary material for: Lipid metabolism characterization in gastric cancer identifies signatures to predict prognostic and therapeutic responses
Source: Front Genet. 2022 Nov 3;13:959170. doi: 10.3389/fgene.2022.959170 (PMC9669965; doi:10.3389/fgene.2022.959170)
Supplement: Supplementary file 4 [file DataSheet1.docx]

**Supplementary Table**

**Table S1.** Clinicopathological information of the GC cohort.

| **Clinicopathological characteristics** | **Number of patients (n = 337)** |
| --- | --- |
| **TCGA-subtype** |  |
| CIN | 197 |
| EBV | 31 |
| GS | 45 |
| HM-SNV | 3 |
| MSI | 59 |
| NA | 32 |
| **Gender** |  |
| Female | 128 |
| Male | 239 |
| **Age** |  |
| < 50 | 59 |
| 50 ~ 70 | 181 |
| >70 | 124 |
| NA | 3 |
| **Race** |  |
| Asian | 74 |
| Black or African American | 11 |
| White | 230 |
| Native Hawaiian or other pacific islander | 1 |
| NA | 51 |
| **OS** |  |
| 0 | 216 |
| 1 | 151 |
| Unknown | NA |
| **Histologic grade** |  |
| Grade 1 | 21 |
| Grade 2 | 121 |
| Grade 3 | 142 |
| Grade 4 | 38 |
| NA | 15 |
| **TNM stage** |  |
| I | 18 |
| II | 85 |
| III | 168 |
| IV | 92 |
| Unknown | 4 |

Compared with the control group, ^*^*p* < 0.05.

**Table S2.** List of mass spectrometric parameters and retention time of 90 MRM pairs (including 6 IS), intraday and interday precision of 84 detected endogenous sphingolipids.

| **Sphingolipid**  **subcategory** | **Sphingolipid**  **species** | **Precursor ion (m/z)** | **Product ion**  **(m/z)** | **Fragmentor**  **(V)** | **CE**  **(V)** | **Rt**  **(min)** | **Intraday CV**  **(%)** | **Interday CV**  **(%)** |
| --- | --- | --- | --- | --- | --- | --- | --- | --- |
| Sph | sphingosine(d17:1) (IS) | 286.3 | 268.3 | 100 | 5 | 1.8 | 2.2 | 11.6 |
|  | sphingosine(d18:1) | 300.3 | 282.2 | 100 | 5 | 2.0 | 2.2 | 11.6 |
| dHCer | Cer (d18:0/12:0) (IS) | 484.5 | 266.4 | 180 | 30 | 5.7 | 2.2 | 11.6 |
|  | Cer (d18:0/16:0) | 540.5 | 266.4 | 180 | 30 | 6.9 | 6.3 | 8.2 |
|  | Cer (d18:0/18:0) | 568.6 | 266.3 | 180 | 30 | 7.6 | 9.5 | 12.6 |
|  | Cer (d18:0/22:0) | 624.6 | 266.3 | 180 | 30 | 8.9 | 6.3 | 10.4 |
|  | Cer (d18:0/23:0) | 638.6 | 266.3 | 180 | 30 | 9.1 | 5.5 | 11.0 |
|  | Cer (d18:0/24:1) | 650.7 | 266.3 | 180 | 30 | 8.9 | 4.8 | 10.4 |
| Cer | Cer (d18:1/17:0) (IS) | 552.5 | 264.3 | 140 | 25 | 7.0 | 2.2 | 11.6 |
|  | Cer (d16:1/22:0) | 594.3 | 236.3 | 140 | 25 | 8.1 | 5.5 | 5.8 |
|  | Cer (d18:1/14:0) | 510.5 | 264.4 | 140 | 25 | 5.9 | 6.9 | 9.5 |
|  | Cer (d18:1/16:0) | 538.5 | 264.4 | 140 | 25 | 6.6 | 3.2 | 4.8 |
|  | Cer (d18:1/18:0) | 566.5 | 264.3 | 140 | 25 | 7.3 | 5.5 | 6.6 |
|  | Cer (d18:1/19:0) | 580.6 | 264.3 | 140 | 25 | 7.7 | 19.9 | 19.9 |
|  | Cer (d18:1/20:0) | 594.6 | 264.3 | 140 | 25 | 8.0 | 4.0 | 4.4 |
|  | Cer (d18:1/21:0) | 608.6 | 264.3 | 140 | 25 | 8.3 | 5.7 | 10.8 |
|  | Cer (d18:1/22:0) | 622.6 | 264.3 | 140 | 25 | 8.7 | 2.7 | 5.3 |
|  | Cer (d18:1/23:0) | 636.6 | 264.3 | 140 | 25 | 9.0 | 5.2 | 10.8 |
|  | Cer (d18:1/23:1) | 634.6 | 264.3 | 140 | 25 | 8.3 | 6.7 | 10.3 |
|  | Cer (d18:1/24:0) | 650.6 | 264.3 | 140 | 25 | 9.3 | 4.4 | 9.2 |
|  | Cer (d18:1/24:1) | 648.6 | 264.3 | 140 | 25 | 8.6 | 3.0 | 4.9 |
|  | Cer (d18:1/25:0) | 664.7 | 264.3 | 140 | 25 | 9.6 | 5.0 | 14.8 |
|  | Cer (d18:1/26:0) | 678.7 | 264.3 | 140 | 25 | 9.8 | 5.5 | 16.6 |
|  | Cer (d18:1/26:1) | 676.7 | 264.3 | 140 | 25 | 9.2 | 5.6 | 8.2 |
|  | Cer (d18:2/16:0) | 536.5 | 262.4 | 140 | 25 | 6.0 | 3.7 | 5.4 |
|  | Cer (d18:2/18:0) | 564.5 | 262.4 | 140 | 25 | 6.8 | 2.9 | 6.0 |
|  | Cer (d18:2/20:0) | 592.6 | 262.3 | 140 | 25 | 7.5 | 7.9 | 8.0 |
|  | Cer (d18:2/21:0) | 606.6 | 262.3 | 140 | 25 | 7.8 | 12.0 | 15.0 |
|  | Cer (d18:2/22:0) | 620.6 | 262.3 | 140 | 25 | 8.2 | 4.0 | 4.9 |
|  | Cer (d18:2/23:0) | 634.6 | 262.3 | 140 | 25 | 8.5 | 4.4 | 8.9 |
|  | Cer (d18:1/20:0) | 594.6 | 264.3 | 140 | 25 | 8.0 | 4.0 | 4.4 |
|  | Cer (d18:1/21:0) | 608.6 | 264.3 | 140 | 25 | 8.3 | 5.7 | 10.8 |
|  | Cer (d18:1/22:0) | 622.6 | 264.3 | 140 | 25 | 8.7 | 2.7 | 5.3 |
|  | Cer (d18:1/23:0) | 636.6 | 264.3 | 140 | 25 | 9.0 | 5.2 | 10.8 |
|  | Cer (d18:1/23:1) | 634.6 | 264.3 | 140 | 25 | 8.3 | 6.7 | 10.3 |
|  | Cer (d18:2/23:1) | 632.6 | 262.3 | 140 | 25 | 7.8 | 20.3 | 20.3 |
|  | Cer (d18:2/24:0) | 648.6 | 262.3 | 140 | 25 | 8.8 | 3.5 | 7.9 |
|  | Cer (d18:2/24:1) | 646.6 | 262.3 | 140 | 25 | 8.1 | 4.4 | 5.8 |
|  | Cer (d18:2/24:2) | 644.4 | 262.3 | 140 | 25 | 7.6 | 7.7 | 7.7 |
| HexCer | GlcCer(d18:1/12:0) (IS) | 644.5 | 264.3 | 150 | 30 | 4.8 | 6.6 | 10.2 |
|  | HexCer (d16:1/22:0) | 756.7 | 236.3 | 150 | 30 | 7.6 | 6.6 | 10.2 |
|  | HexCer (d18:1/14:0) | 672.6 | 264.3 | 150 | 30 | 5.4 | 5.8 | 9.7 |
|  | HexCer (d18:1/16:0) | 700.6 | 264.3 | 150 | 30 | 6.1 | 2.2 | 3.8 |
|  | HexCer (d18:1/18:0) | 728.6 | 264.3 | 150 | 30 | 6.8 | 3.9 | 5.7 |
|  | HexCer (d18:1/19:0) | 742.6 | 264.3 | 150 | 30 | 7.2 | 15.4 | 18.8 |
|  | HexCer (d18:1/20:0) | 756.6 | 264.3 | 150 | 30 | 7.5 | 5.6 | 5.6 |
|  | HexCer (d18:1/22:0) | 784.7 | 264.3 | 150 | 30 | 8.2 | 3.2 | 4.8 |
|  | HexCer (d18:1/23:0) | 798.7 | 264.3 | 150 | 30 | 8.5 | 3.1 | 7.7 |
|  | HexCer (d18:1/24:0) | 812.7 | 264.3 | 150 | 30 | 8.8 | 2.5 | 8.6 |
|  | HexCer (d18:1/24:1) | 810.7 | 264.3 | 150 | 30 | 8.2 | 3.1 | 5.4 |
|  | HexCer (d18:1/25:0) | 826.7 | 264.3 | 150 | 30 | 9.1 | 3.2 | 12.6 |
|  | HexCer (d18:1/26:1) | 838.7 | 264.3 | 150 | 30 | 8.8 | 11.1 | 11.3 |
|  | HexCer (d18:2/16:0) | 698.6 | 262.3 | 150 | 30 | 5.5 | 5.9 | 6.7 |
|  | HexCer (d18:2/18:0) | 726.6 | 262.3 | 150 | 30 | 6.2 | 9.5 | 10.7 |
|  | HexCer (d18:2/20:0) | 754.6 | 262.3 | 150 | 30 | 6.9 | 8.0 | 8.8 |
|  | HexCer (d18:2/22:0) | 782.7 | 262.3 | 150 | 30 | 7.7 | 4.8 | 5.7 |
|  | HexCer (d18:2/23:0) | 796.7 | 262.3 | 150 | 30 | 8.0 | 8.4 | 9.1 |
|  | HexCer (d18:2/24:0) | 810.7 | 262.3 | 150 | 30 | 8.3 | 1.7 | 5.9 |
|  | HexCer (d18:2/26:0) | 838.7 | 262.3 | 150 | 30 | 9.0 | 13.3 | 20.1 |
| LacCer | LacCer(d18:1/12:0) (IS) | 806.5 | 264.3 | 190 | 45 | 4.6 |  |  |
|  | LacCer (d18:1/16:0) | 862.6 | 264.3 | 190 | 45 | 5.9 | 5.4 | 6.8 |
|  | LacCer (d18:1/18:1) | 888.6 | 264.3 | 190 | 45 | 6.1 | 5.6 | 6.8 |
|  | LacCer (d18:1/24:1) | 972.7 | 264.3 | 190 | 45 | 7.9 | 6.1 | 7.8 |
|  | LacCer (d18:2/16:0) | 860.6 | 262.3 | 190 | 45 | 5.3 | 3.6 | 3.6 |
|  | LacCer (d18:2/24:1) | 970.7 | 262.3 | 190 | 45 | 7.4 | 6.8 | 6.9 |
| SM | SM(d18:1/12:0) (IS) | 647.5 | 184 | 150 | 20 | 4.6 | - | - |
|  | SM32:0 | 677.5 | 184 | 150 | 20 | 5.4 | 1.6 | 4.2 |
|  | SM32:1 | 675.5 | 184 | 150 | 20 | 5.2 | 1.5 | 4.8 |
|  | SM33:0 | 691.5 | 184 | 150 | 20 | 5.7 | 4.7 | 5.0 |
|  | SM33:1 | 689.6 | 184 | 150 | 20 | 5.5 | 2.2 | 2.6 |
|  | SM34:0 | 705.6 | 184 | 150 | 20 | 6.1 | 1.8 | 5.7 |
|  | SM34:1 | 703.6 | 184 | 150 | 20 | 5.9 | 3.5 | 5.6 |
|  | SM34:2 | 701.6 | 184 | 150 | 20 | 5.3 | 1.2 | 2.4 |
|  | SM34:2-OH | 717.6 | 184 | 150 | 20 | 5.1 | 3.4 | 3.8 |
|  | SM36:0 | 733.6 | 184 | 150 | 20 | 6.8 | 4.8 | 8.0 |
|  | SM36:1 | 731.6 | 184 | 150 | 20 | 6.6 | 3.3 | 7.3 |
|  | SM36:2 | 729.6 | 184 | 150 | 20 | 6.0 | 1.9 | 5.4 |
|  | SM36:3 | 727.6 | 184 | 150 | 20 | 5.5 | 2.3 | 3.0 |
|  | SM37:1 | 745.6 | 184 | 150 | 20 | 7.0 | 2.8 | 7.7 |
|  | SM38:1 | 759.6 | 184 | 150 | 20 | 7.3 | 3.9 | 6.7 |
|  | SM38:2 | 757.6 | 184 | 150 | 20 | 6.7 | 2.9 | 8.0 |
|  | SM39:1 | 773.7 | 184 | 150 | 20 | 7.7 | 3.1 | 5.3 |
|  | SM39:2 | 771.6 | 184 | 150 | 20 | 7.1 | 3.9 | 5.7 |
|  | SM40:1 | 787.7 | 184 | 150 | 20 | 8.0 | 3.8 | 6.2 |
|  | SM40:2 | 785.7 | 184 | 150 | 20 | 7.5 | 3.6 | 6.0 |
|  | SM40:3 | 783.6 | 184 | 150 | 20 | 6.7 | 3.3 | 9.6 |
|  | SM41:1 | 801.7 | 184 | 150 | 20 | 8.3 | 2.6 | 4.2 |
|  | SM41:2 ***** | 799.7 | 184 | 150 | 20 | 7.6, 7.8 | 2.9 | 4.5 |
|  | SM41:3 | 797.6 | 184 | 150 | 20 | 7.1 | 3.1 | 7.0 |
|  | SM42:1 | 815.7 | 184 | 150 | 20 | 8.6 | 2.7 | 4.2 |
|  | SM42:2 ***** | 813.7 | 184 | 150 | 20 | 7.9, 8.2 | 3.2 | 5.9 |
|  | SM42:3 | 811.7 | 184 | 150 | 20 | 7.4 | 3.9 | 5.5 |
|  | SM42:4 | 809.7 | 184 | 150 | 20 | 6.8 | 2.2 | 7.4 |
|  | SM42:5 | 807.6 | 184 | 150 | 20 | 6.5 | 2.3 | 6.8 |
|  | SM43:1 | 829.7 | 184 | 150 | 20 | 9.0 | 2.0 | 6.5 |

*, SM isomers sharing identical total carbon number and unsaturation but differing in sphingoid base backbone were separated by RPLC and further identified by MS/MS. Isomers of SM41:2 eluting at 7.6 and 7.8 min were SM (d17:1/24:1) and SM (d18:2/23:0); isomers of SM42:2 eluting at 7.9 and 8.2 min were SM (d18:1/24:1) and SM (d18:2/24:0).

**Table S3.** Summary of mass spectrometric parameters and number of endogenous sphingolipids detected by untargeted metabolomics method.

| **Sphingolipids** | **mass spectrometric parameters** | | | | **Corresponding IS** | **Endogenous species detected** |
| --- | --- | --- | --- | --- | --- | --- |
|  | **precursor**  **(m/z)** | **product**  **(m/z)** | **fragmentor**  **(V)** | **CE**  **(V)** |  |  |
| Sphingosine | 300.3 | 282.2 | 100 | 5 | Sphingosine(d17:1) | 1 |
| Ceramide | [M+H]+ | 264.3/262.3/236.3^*^ | 140 | 25 | Cer(d18:1/17:0) | 25 |
| Dihydroceramide | [M+H]+ | 266.3 | 180 | 30 | Cer(d18:0/12:0) | 5 |
| Hexosylceramide | [M+H]+ | 264.3/262.3/236.3^*^ | 150 | 30 | GlcCer(d18:1/12:0) | 19 |
| Lactosylceramide | [M+H]+ | 264.3/262.3^*^ | 190 | 45 | LacCer(d18:1/12:0) | 5 |
| Sphingomyelin | [M+H]+ | 184 | 150 | 20 | SM(d18:1/12:0) | 29 |

^*^, Product ions 264,262,236 indicate backbone of sphingoid base d18:1,d18:2 and d16:1, respectively. CE means collision energy.

**Supplementary Figure Legends**

**Figure S1**. Validation of high (n = 91) and low (n = 91) LMscore groups from the GSE15459 cohort by Kaplan-Meier curves. Log-rank test shows an overall *p* = 0.0052.

**Figure S2**. LMscore differences in the clinicopathological characteristic subtypes. **A**, T stage; **B**, Age; **C**, Grade; **D**, Stage; **E**, Lymph node.

**Figure S3**. Validation of the robustness of LMscore differences in the clinicopathological characteristic subtypes.
